# Indomethacin Disrupts Autophagic Flux by Inducing Lysosomal Dysfunction in Gastric Cancer Cells and Increases Their Sensitivity to Cytotoxic Drugs

**DOI:** 10.1038/s41598-018-21455-1

**Published:** 2018-02-26

**Authors:** Jorge Vallecillo-Hernández, Maria Dolores Barrachina, Dolores Ortiz-Masiá, Sandra Coll, Juan Vicente Esplugues, Sara Calatayud, Carlos Hernández

**Affiliations:** 1 Departamento de Farmacología and CIBERehd, Facultad de Medicina, Universidad de Valencia, Av. Blasco Ibáñez, 15, 46010 Valencia, Spain; 2 Departamento de Medicina and CIBERehd, Facultad de Medicina, Universidad de Valencia, Av. Blasco Ibáñez, 15, 46010 Valencia, Spain; 3 FISABIO, Hospital Dr. Peset, Av. Cataluña, 21, 46020 Valencia, Spain

## Abstract

NSAIDs inhibit tumorigenesis in gastrointestinal tissues and have been proposed as coadjuvant agents to chemotherapy. The ability of cancer epithelial cells to adapt to the tumour environment and to resist cytotoxic agents seems to depend on rescue mechanisms such as autophagy. In the present study we aimed to determine whether an NSAID with sensitizing properties such as indomethacin modulates autophagy in gastric cancer epithelial cells. We observed that indomethacin causes lysosomal dysfunction in AGS cells and promotes the accumulation of autophagy substrates without altering mTOR activity. Indomethacin enhanced the inhibitory effects of the lysosomotropic agent chloroquine on lysosome activity and autophagy, but lacked any effect when both functions were maximally reduced with another lysosome inhibitor (bafilomycin B1). Indomethacin, alone and in combination with chloroquine, also hindered the autophagic flux stimulated by the antineoplastic drug oxaliplatin and enhanced its toxic effect, increasing the rate of apoptosis/necrosis and undermining cell viability. In summary, our results indicate that indomethacin disrupts autophagic flux by disturbing the normal functioning of lysosomes and, by doing so, increases the sensitivity of gastric cancer cells to cytotoxic agents, an effect that could be used to overcome cancer cell resistance to antineoplastic regimes.

## Introduction

Epithelial cells of the gastrointestinal system are targets for non-steroidal anti-inflammatory drugs (NSAIDs). When used to treat pain and inflammation these drugs exert a deleterious effect on digestive epithelia, which constitutes their main side effect. However, this activity of NSAIDs has a knock-on positive effect by inhibiting tumorigenesis in gastrointestinal tissues, the underlying mechanisms of which are poorly characterized^1^. Additionally, NSAIDs have been tested as coadjuvants to antineoplastic regimes, with promising results obtained^2–6^. Both the abovementioned gastrointestinal toxicity and potential antineoplastic effect seem to be related with a reduction in the ability of epithelial cells of either normal or cancerous origin, to circumvent the damaging effects of aggressive digestive juices in the former case and scarcity of nutrients and cytotoxic treatments in the latter. In cells exposed to such challenging situations, the occurrence or avoidance of apoptosis often depends on the activation of rescue mechanisms like macroautophagy (hereafter referred to as autophagy)^7^. In fact, recent evidence suggests that the resistance to some cytotoxic agents is subject to the activation of autophagy^8^.

The objective of autophagy is to degrade superfluous and damaged organelles, cytosolic proteins and invasive microbes by forming a double-membrane sequestering compartment termed the phagophore, which matures into an autophagosome. Once the cargo has been delivered to the lysosome and degraded, the resulting macromolecules are released back into the cytosol and used as macromolecular constituents and energy sources in order to maintain cell viability, thus constituting the predominant role of autophagy^7^. Indeed, in the case of aspirin, we have observed that inhibition of this process contributes to the drug’s gastrotoxicity^9^. However, autophagy has also been implicated in cell death, and recent studies have linked it to the deleterious action of another classical NSAID, indomethacin, in primary gastric^10^ and intestinal cells^11^. Taking into consideration that indomethacin has shown potential as a sensitizing agent with regard to the cytotoxic effects of anticancer drugs^12–15^, in the present study we aimed to determine the effects of this NSAID on autophagy in gastric cancer epithelial cells and how they influence cell sensitivity to an antineoplastic agent.

## Results

### Indomethacin inhibits autophagic degradation in AGS cells

**Figure 1:**
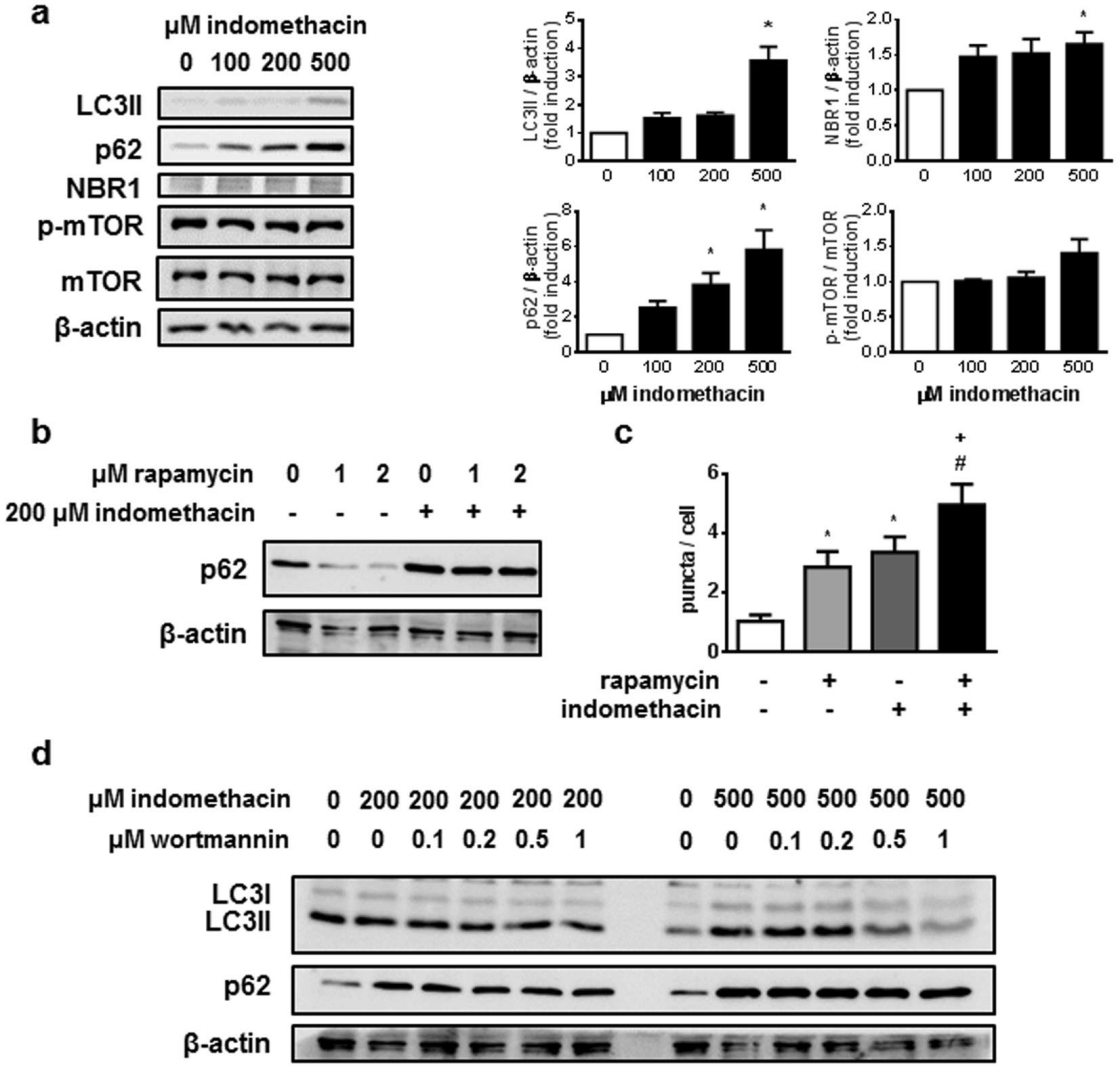
Indomethacin inhibits autophagy degradation in AGS cells. (**a**) Representative Western blots for LC3, p62, NBR1, phosphorylated mTOR at Ser2481, total mTOR and actin from cells treated with increasing doses of indomethacin or vehicle. Graphs represent relative densitometric quantification performed using the Multi Gauge software (Fujifilm) (n = 6). **(b)** Representative Western blots for p62 and actin from cells treated with 200 µM indomethacin and rapamycin (1 and 2 µM) (n = 3). **(c)** Number of LC3 positive dots per cell in AGS cells stably expressing the p3xFLAG/EmGFP/LC3B construct and treated with 200 µM indomethacin. Cells were incubated with vehicle or 1 µM rapamycin for the last 2 hours (n = 4) **(d)** Representative Western blots for LC3, p62 and actin from cells treated with 200 and 500 µM indomethacin and increasing doses of wortmannin (n = 3). Indomethacin treatment period was 24 hours in all cases. Data in figures represent mean ± SEM. *P < 0.05 vs control, ^+^P < 0.05 vs indomethacin, and ^#^P < 0.05 vs rapamycin groups (ANOVA and Newman-Keuls).

First, we determined protein levels of several autophagic markers (LC3, p62 and NBR1) in AGS cells after 24-hour treatment with indomethacin. The LC3-I cytosolic form is transformed by lipidation on the autophagosome component LC3-II which, once this vesicle has fused with the lysosome, is degraded or recycled. LC3-II protein levels in AGS cells were increased by indomethacin, which may have been a consequence of either induction of autophagy or inhibition of lysosomal-dependent autophagic degradation (Fig. 1a).

The p62 and NBR1 proteins are autophagy receptors that attract specific autophagy targets to the autophagosome by interacting with them and with LC3-II protein. Eventually enclosed within the autophagosome/autolysosome, they are degraded by lysosomal proteases. Treatment of AGS cells with indomethacin resulted in the accumulation of both p62 and NBR1 proteins (Fig. 1a), suggesting that this drug inhibits lysosomal degradation of autophagic targets. Indomethacin induced a similar increase in LC3-II and p62 protein levels in colonic epithelial cells (HT29 cells) (Supplementary Figure S1).

The PI3K/Akt/mTOR pathway is an essential regulator of autophagy that inhibits its activation under nutrient-rich conditions. Treatment with indomethacin does not significantly modify the autophosphorylation of mTOR at Ser 2481 (Fig. 1a), indicating that inhibition of autophagic degradation is not due to inhibition of mTOR, and therefore should occur downstream from this regulator. This idea is further substantiated by the fact that indomethacin blocked the degradation of p62 induced by the mTOR inhibitor rapamycin (Fig. 1b**)**, while the indomethacin-induced accumulation of LC3-II, but not of p62, was reduced when cells were treated in the presence of wortmannin, a general PI3K inhibitor whose action downstream from mTOR is capable of inhibiting basal autophagy (Fig. 1d). Moreover, in AGS cells stably expressing a fusion protein containing an EmGFP tag in tandem with the LC3B protein to identify and quantify LC3-II-positive dots, we observed an increased number of LC3-II puncta in cells treated with either the autophagy stimulator rapamycin or with indomethacin. This increase was significantly enhanced when cells were treated with both drugs, which suggests that indomethacin blocks the degradation of autophagosomes that are newly formed in response to rapamycin (Fig. 1c).

### Indomethacin impairs lysosomal function in AGS cells

**Figure 2:**
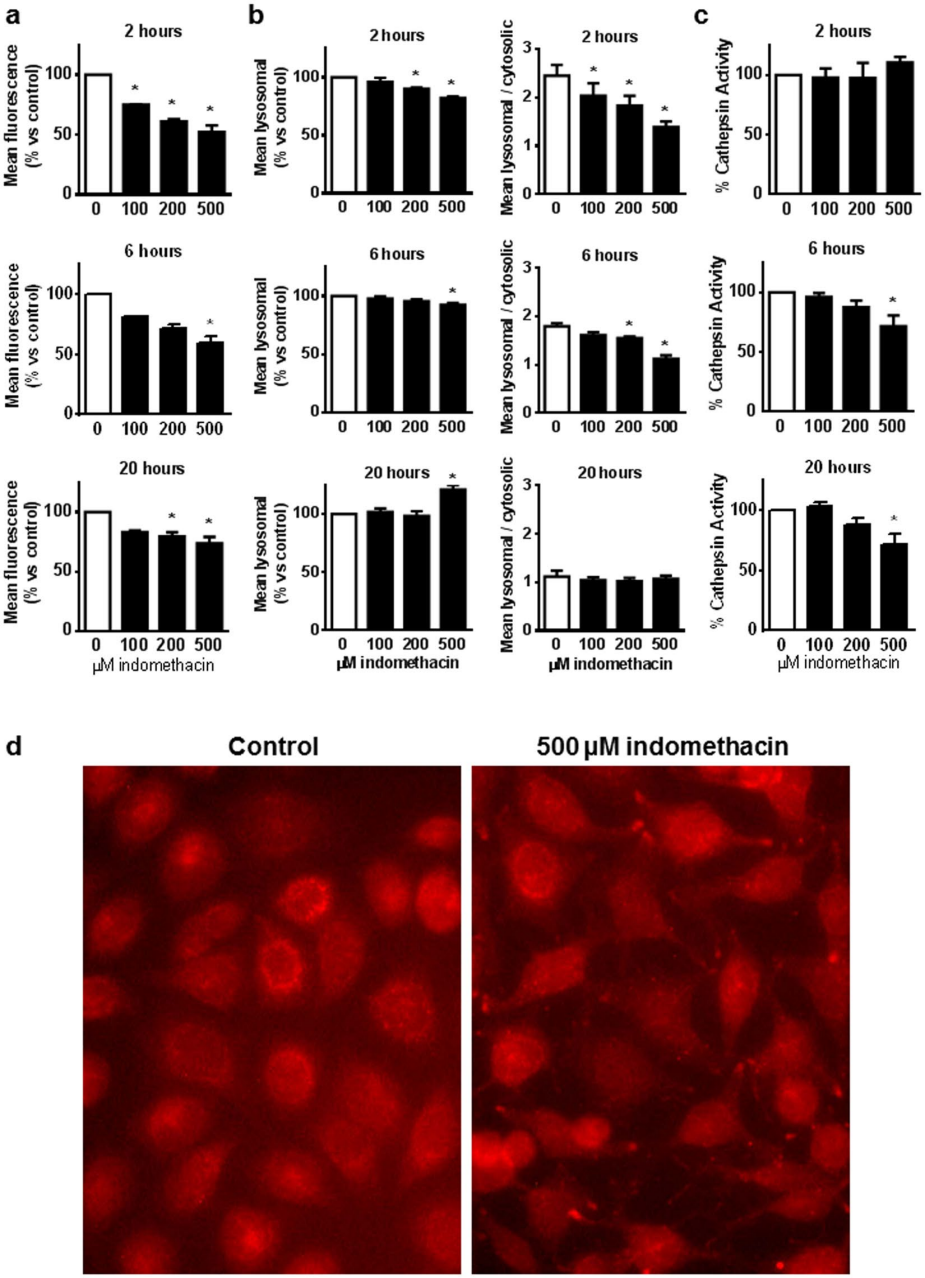
Indomethacin impairs lysosomal function in AGS cells. Cells were treated with increasing doses of indomethacin or vehicle and the following analysis was performed 2, 6 and 20 hours later: **(a)** LysoTracker® Red DND99 fluorescence measured by static cytometry (n = 4). **(b)** Acridine Orange red (lysosomal) fluorescence (left panels) and ratio between red (lysosomal)/green (cytosolic) fluorescence (right panels) measured by static cytometry (n = 4). **(c)** AMC fluorescence in cell lysates as an index of the enzymatic activity of cathepsins acting on the Z-FR-AMC substrate (n = 4). **(a**–**c)** Data represent mean ± SEM, *P < 0.05 by ANOVA and Newman-Keuls. **(d)** Representative photographs of immunocytochemistry for LAMP2 performed in cells treated with 500 µM indomethacin or vehicle for 2 hours (n = 3).

We investigated the impact of indomethacin on lysosomes, as they are the final destination of autophagic cargoes. Their degrading activity depends on the maintenance of an acidic pH in the lumen that allows lysosomal enzymes to exert their activity. The effects of indomethacin treatment on lysosomal pH were first analyzed using the acidophilic probe Lysotracker. Indomethacin decreased Lysotracker fluorescence in AGS cells after 2, 6 and 20 hours of treatment (Fig. 2a), suggesting that the drug reduces Lysotracker accumulation in lysosomes. In a second set of experiments, we used acridine orange, which labels lysosomes with red fluorescence and the cytosol with green fluorescence, depending on pH conditions. Again, indomethacin caused an acute reduction in lysosome-derived fluorescence (2 and 6 hours), although an increased signal was observed after 20 hours (Fig. 2b). In addition, the ratio between cytosolic and lysosomal fluorescence, suggestive of lysosome membrane permeability, was also acutely (2 and 6 h) reduced and later normalized (20 h) (Fig. 2b). Similar results were obtained in HT29 cells (Supplementary Figure S1). In order to determine whether these alterations were due to a reduction of the total lysosomal compartment rather than specific changes in lysosomal conditions, we analyzed LAMP2 staining in AGS cells treated with indomethacin for 2 hours, observing that, in the absence of quantitative changes in LAMP2 expression (data not shown), there was an altered distribution of LAMP2-positive dots from the perinuclear position observed in control cells to a peripheral position in treated cells (Fig. 2d). This re-distribution has recently been related with an increased luminal pH in lysosomes. Therefore, taken as a whole, our data indicate that indomethacin reduces lysosomal acidity.

In addition, we measured cathepsin activity using a fluorogenic substrate cleaved by most cathepsins (Z-FR-AMC), and observed a significant reduction of this enzymatic activity in cells treated with indomethacin for 6 or 20 hours (Fig. 2c). This suggests that, by increasing lysosomal pH, indomethacin inhibits the activity of acidic lysosomal enzymes.

### Differential effects of indomethacin on lysosomal function upon chloroquine and bafilomycin treatment

**Figure 3:**
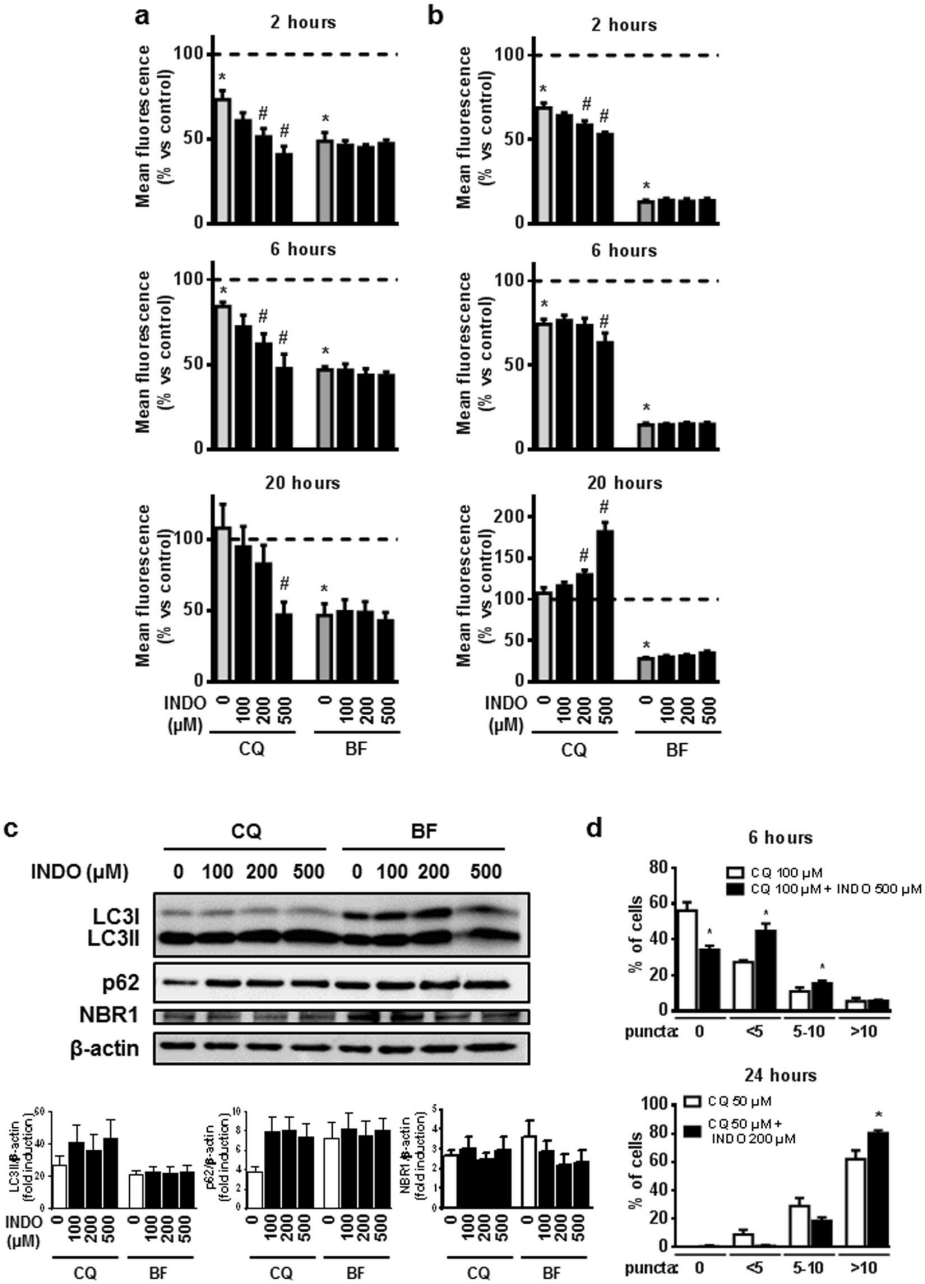
Chloroquine and bafilomycin B1 modulate the effects of indomethacin. AGS cells were treated with increasing doses of indomethacin (INDO) in the presence or absence of chloroquine (CQ, 100 µM for 2 and 6 hours, 50 µM for 20 hours), or bafilomycin B1 (BF, 50 nM for 2, 6 and 20 hours). After treatment, cells were incubated with LysoTracker® Red DND99 **(a)**, or Acridine Orange **(b)**, and lysosomal fluorescence was measured by static cytometry (n = 4 in all cases). **(c)** Representative Western blots for LC3, p62, NBR1, and actin from cells treated with increasing doses of indomethacin in the presence of chloroquine (50 µM) or bafilomycin B1 (50 nM) for 24 hours. Graphs show relative densitometric quantification performed using the Multi Gauge software (Fujifilm) (n = 6). **(d)** Percentage of p3xFLAG/EmGFP/LC3B-AGS cells with different amounts of LC3 positive dots after treatment with indomethacin and chloroquine for 6 and 24 hours (n = 3). In all graphs, data represent mean ± SEM. *P < 0.05 vs. control, ^#^P < 0.05 vs respective inhibitor (chloroquine or bafilomycin) (ANOVA and Newman-Keuls).

To further examine the role of lysosomes in the actions of indomethacin, we analyzed the effects of the NSAID in the presence of two different lysosome inhibitors: chloroquine and bafilomycin B1. Treatment with chloroquine alone induced a slight reduction in Lysotracker fluorescence (Fig. 3a) and lysosomal-derived acridine orange fluorescence (Fig. 3b). This effect was maximal, since it did not increase with a higher dose of chloroquine (200 µM) that, conversely, produced enhanced toxicity (data not shown). When chloroquine was combined with indomethacin a further reduction in these parameters was observed. Moreover, the effect of indomethacin on the distribution of LAMP2-positive dots towards the periphery of the cells was sustained when chloroquine was present (data not shown), suggesting that this indomethacin-induced mechanism complements the inhibition of lysosomal function by chloroquine. In contrast, bafilomycin B1 consistently decreased both parameters and indomethacin did not exert further actions on this effect (Fig. 3a and b). Similarly, in HT29 cells, indomethacin also reduced Lysotracker fluorescence in the presence of chloroquine and did not modify either Lysotracker or acridine orange fluorescence in the presence of bafilomycin B1 (Supplementary Figure S1). These data indicate differences between the two lysosomal inhibitors that were also patent in their effects on autophagic degradation. Indomethacin was not able to increase LC3-II and p62 accumulation in cells treated with bafilomycin B1. However, in the presence of chloroquine, it induced a non-significant increase in the levels of these proteins in AGS cells (Fig. 3c) and HT29 cells (Supplementary Figure S1), together with a significant accumulation of LC3-II puncta in AGS cells stably expressing EmGFP-LC3B (Fig. 3d, Supplementary Figure S2).

Static cytometry performed at the end of the treatment period revealed that indomethacin had produced a dose- and time- dependent toxicity, manifested as a progressive reduction in the number of cells per field. This toxicity was enhanced by co-treatment with lysosome inhibitors, especially with chloroquine, the doses of which were reduced as the treatment period was extended in order to perform the analysis in comparable circumstances (Supplementary Figure S3).

### Indomethacin increases oxaliplatin-induced cell death in AGS cells

**Figure 4:**
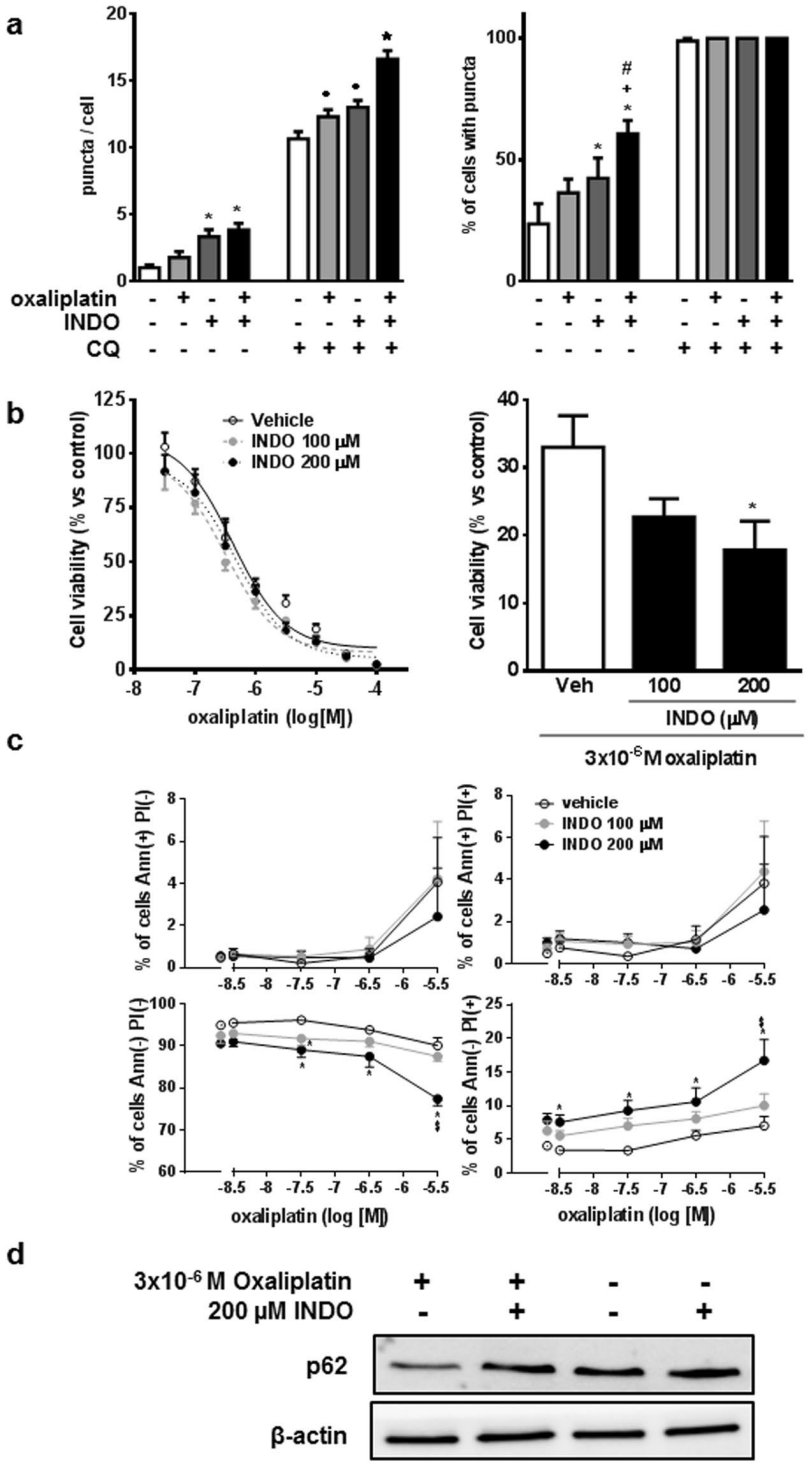
Indomethacin enhances oxaliplatin-induced cell death of AGS cells. **(a)** Number of LC3 positive dots per cell (left panel) and percentage of cells with puncta (right panel) in p3xFLAG/EmGFP/LC3B-AGS cells treated for 24 hours with 200 µM indomethacin (INDO) and/or 30 µM oxaliplatin in the absence or presence of chloroquine (CQ) 50 µM (n = 4). **(b–d)** AGS cells treated with increasing doses of oxaliplatin for 48 hours plus vehicle and 100 and 200 µM indomethacin for the last 24 hours were analyzed with regard to: **(b)** Cell viability, assessed by MTT assay (n = 6, curve comparison by two-way ANOVA: P = 0.0146), **(c)** AnnexinV-fluorescein binding to exteriorized phosphatidylserine in apoptotic cells and propidium iodide permeation in dead or damaged cells, assessed by static cytometry (n = 5); and **(d)** p62 and actin levels, measured by Western blot (images representative of n = 3). In all graphs, data represent mean ± SEM. *P < 0.05 vs control, ^+^P < 0.05 vs indomethacin, ^#^P < 0.05 vs oxaliplatin, ^•^P < 0.05 vs chloroquine, ^★^P < 0.05 vs all chloroquine-treated groups and ^&^P < 0.05 vs indomethacin 100 µM (ANOVA and Newman-Keuls).

To investigate whether the influence of indomethacin on autophagy affects the cytotoxicity of oxaliplatin, an antineoplastic drug commonly used to treat gastric cancer, we measured the effect of a combination of both drugs on AGS cells. In control conditions oxaliplatin tended to increase the number of LC3-II dots in AGS cells stably expressing EmGFP-LC3B, an effect that was enhanced in the presence of the lysosome inhibitor chloroquine (Fig. 4a). In both circumstances, the accumulation of autophagosomes was more pronounced when cells were also treated with indomethacin. An MTT assay revealed that oxaliplatin decreased cell viability in a dose-dependent manner after 48 hours of treatment, and treatment of cells with indomethacin during the last 24 hours further decreased cell viability (Fig. 4b). In addition, indomethacin also increased the rate of necrosis in AGS cells treated with oxaliplatin (Fig. 4c). The effects on cell death correlated with the inhibition by indomethacin of the increased p62 degradation observed in oxaliplatin-treated AGS (Fig. 4d) and HT29 (Supplementary Figure S1) cells. These results suggest that treatment of cancer cells with indomethacin blocks the activation of autophagic flux by oxaliplatin and increases the sensitivity to the cytotoxic action of the latter drug.

## Discussion

The present study demonstrates that indomethacin sensitizes cancer cells to the toxic action of chemotherapy agents and that this action is related to deficient autophagy caused by an inhibitory effect on lysosomes, organelles with apparent potential as targets for chemotherapy^16,17^.

Indomethacin-treated cells presented higher amounts of three different autophagy substrates - the autophagosome membrane component LC3-II, and the cargo receptors p62 and NBR1 - suggesting that indomethacin inhibits the autophagic degradation process. Indomethacin seemed to disrupt a late step in the autophagic route, since the substrates accumulated without altering the activity of the master regulator of autophagy mTOR and continued to accumulate when autophagy was stimulated by the mTOR inhibitor rapamycin. Additionally, levels of the phagophore component LC3-II were reduced when autophagic flux downstream from mTOR was inhibited by the PI3K blocker wortmannin. We tested the lysosomal function of indomethacin-treated cells and observed a reduction in the fluorescent signal emitted by two different acidophilic probes, Lysotracker and acridine orange, which was indicative of lysosomal basification. The reduced ratio between the red-lysosomal and the green-cytoplasmic fluorescent signal emitted by acridine orange in indomethacin-treated cells also suggested increased lysosomal membrane permeability. Interestingly, these changes were associated with a centrifugal movement of lysosomes from their normal perinuclear position to the cell periphery, which has recently been linked to reduced acidity and increased permeability, as observed in the present study^18,19^. Consistent with the alkalinisation of lysosomes, we observed a reduced activity of cathepsins in indomethacin-treated cells, which explains the deficient degradation of autophagic substrates.

To further analyze the role of the lysosomal actions of indomethacin on autophagic inhibition we evaluated its effects in the presence of two lysosomal inhibitors: bafilomycin B1 and chloroquine. The first possesses a well-characterized dual mechanism of action involving the blockade of both the V-ATPase responsible for lysosomal acidification and lysosome-autophagosome fusion^7^. In our experiments, bafilomycin B1 induced a significant inhibition of all lysosomal parameters and logically caused the accumulation of all three autophagic substrates (LC3-II, p62 and NBR1). None of these effects was enhanced by co-treatment with indomethacin, which suggests that the disruption of lysosomal function by bafilomycin outweighs the activity of indomethacin and further supports the hypothesis that lysosomes are the target of indomethacin.

The combination of indomethacin and chloroquine appears to generate a more complex situation. Chloroquine induced a mild lysosomal dysfunction that was potentiated by indomethacin. Accordingly, the accumulation of p62 induced by bafilomycin B1 was higher than that induced by chloroquine, but it was equalled by the combination of chloroquine and indomethacin. With regard to LC3-II, the effects of chloroquine were comparable to those induced by bafilomycin B1 and, even so, LC3-II levels tended to be higher in cells treated with the combination of chloroquine and indomethacin. In fact, this combination of drugs resulted in the accumulation of a higher number of LC3-II puncta. Since an activation of autophagic flux as a compensatory response to lysosomal blockade seems unlikely in light of the results obtained with bafilomycin B1 and the unchanged mTOR activity^20^, it is possible that chloroquine, alone or in combination with indomethacin, provokes non-canonical LC3 lipidation^21^, a process that would not occur in the presence of bafilomycin B1 because it depends on V-ATPase activity. Notwithstanding, as a whole, our results indicate that indomethacin potentiates the effects of chloroquine, a drug that is traditionally used against malaria and is undergoing clinical trials as a coadjuvant to antineoplastic regimes^22,23^. In accordance with our results, previous studies report a parallelism between indomethacin and chloroquine with regard to their effects on cancer epithelial cells^24^ and a synergy between the two drugs in their action against malaria^25^.

The rationale for incorporating chloroquine into antineoplastic regimes is based on the fact that autophagy occurs as a mechanism of cancer cell resistance to cytotoxic drugs^22,23^. We have observed that oxaliplatin, an antineoplastic drug commonly used in gastric cancer, tends to increase the presence of LC3-II puncta in AGS cells, and that this increase is more pronounced in the presence of indomethacin, alone or combined with chloroquine. It seems, therefore, that oxaliplatin promotes autophagy in these gastric cancer cells and that indomethacin, alone and especially in the presence of chloroquine, inhibits the degradation of the newly formed autophagosomes. When we added indomethacin to the culture media after a period in the presence of oxaliplatin, this NSAID enhanced the toxic effect of oxaliplatin and produced a concomitant decrease in autophagic activity, which may explain the synergy observed between the two drugs in the present study and previously in an *in vivo* model of cancer^26^. Thus, inhibition of lysosomal function and the consequent interference with autophagic degradation may be partly responsible for the beneficial effects of indomethacin in cancerous processes reported in some studies^4,26,27^.

In contrast to that, which occurs in cancerous gastric epithelial cells, a toxic effect of autophagy has been reported in non-transformed gastric and intestinal cells^10,11,28^. These studies show that inhibition of autophagy by pharmacological or genetic means protected cells against indomethacin’s cytotoxicity and assumed that indomethacin causes damage by stimulating autophagy. However, some of their results challenge the idea of an activated autophagy. Indomethacin-treated cells presented a higher LC3-II/LC3-I ratio and increased levels of LC3-II, total LC3 and ATG5^10,11^, perhaps due to induction of autophagy or inhibition of autophagic degradation. To determine which of the two is responsible, autophagic flux needed to be evaluated in the presence of a lysosomal inhibitor^7^. The only result reported in this respect advocates an inhibitory action, since indomethacin failed to increase the number of autophagosomes in chloroquine-treated cells^10^. This was corroborated by the reported accumulation of ubiquitinated proteins in cells treated with indomethacin^28^. Other studies using the COX-1 inhibitor SC-560^29^ or sulindac^30^ have proposed a stimulatory effect on autophagy without analyzing autophagic flux, which makes them inconclusive. On the other hand, a well-contrasted stimulatory action of the COX-2 inhibitor celecoxib on this process has been observed in several human colorectal cell lines^31^. Thus, the effects of NSAIDs on autophagy in gastrointestinal epithelial cells are far from clear, but would seem to be drug-specific.

Regardless of indomethacin’s impact on autophagic flux, the fact that inhibition of autophagy exerts a protective effect against its deleterious action^10,11,28^ is intriguing. It is possible that the inhibition of autophagic degradation and consequent accumulation of autophagosomes/autolysosomes provoke a toxic effect that is diminished by preventing the formation of these structures, despite an accumulation of autophagic substrates in the cytosol. In line with this argument, different autophagy inhibitors may have varying effects on cell survival depending on the phase (early or late) of the process targeted. This needs to be addressed to reveal further knowledge about the complex relationship between autophagy and cell survival/death, especially in the context of cancer^32,33^. Our results point to the well-known NSAID indomethacin, alone or combined with the classic drug chloroquine, as a candidate drug whose ability to sensitize cancer cells to cytotoxic agents should be tested further.

## Methods

### Cell culture and treatment

AGS cells (ATCC) were cultured in F12K medium (Life Technologies) supplemented with 10% inactivated FBS, 100 U/ml penicillin, 100 μg/ml streptomycin, and 100 mM sodium pyruvate. HT29 cells (ATCC) were cultured in McCoy’s 5 A medium (Life Technologies) supplemented with 10% inactivated FBS, 100 U/ml penicillin, 100 μg/ml streptomycin, and 2 mM glutamine.

In order to obtain cells with a stable expression of a fluorescence-labelled LC3B protein, we transfected AGS cells with the p3xFLAG/EmGFP/LC3B plasmid^9^ using Lipofectamine-2000 (Life Technologies). Following transfection, positive cells were selected by adding 250 µg/mL of the antibiotic G418 (Sigma-Aldrich) to the culture medium. After several passages, cells were sorted with FACSAria III (BD) to obtain a pure culture of green cells, which were maintained in medium supplemented with G418.

For treatment purposes, cells were incubated in culture medium with reduced serum (0.5% of inactivated FBS).

### Protein extraction and Western blot analysis

Cells were homogenized in lysis buffer containing proteases (Complete Mini tablets, Roche) and phosphatase inhibitors. Equal amounts of protein were loaded onto SDS-PAGE gels and analyzed by Western blot (antibodies in Supplementary Table S1). Protein bands were detected with Supersignal chemiluminescent substrate (Thermo Scientific) in a LAS-3000 (Fujifilm). Protein densitometry was performed using the Multi Gauge software (Fujifilm).

### Fluorescence microscopy and immunocytochemistry

AGS cells stably expressing p3xFLAG/EmGFP/LC3B were fixed with formalin after treatment and observed through an inverted microscope (Olympus IX81). Nine photographs (20×) per well were taken and the number of cells with autophagosomes and the amount of LC3-puncta per cell were counted by an observer unaware of the treatment administered.

Immunocytochemistry for LAMP2 was performed in formalin-fixed AGS cells. After blocking with BSA and goat serum, cells were incubated with the primary antibody (overnight, 4 °C) followed by a texas red - conjugated secondary antibody and 5 µM of Hoechst 33342 (45 min, room temperature) (antibodies in Supplementary Table S1). Cells were observed and photographed as described above and were analyzed by static cytometry (see below).

### Static cytometry

Cell fluorescence was measured by means of an automated inverted microscope (Olympus IX81) and Scan^R^ software, which allows cytometric analysis of the signal emitted by individual cells identified as Hoechst 33342 positive events. The number of cells was registered to assess the effect of treatments on cell viability.

For lysosomal pH analysis, AGS cells were incubated for 30 min with LysoTracker® Red DND99 (Life Technologies, 0.1 µM, formalin-fixed cells) or with Acridine Orange (Sigma-Aldrich, A8097; 5 µM, non-fixed cells). LAMP2 expression was detected as described above.

The rate of apoptosis/necrosis was analyzed by means of the Apoptosis Detection Kit (Abcam), which detects phosphatidylserine exteriorization in apoptotic cells with AnnexinV-fluorescein and cell permeability to the chromatin-detecting dye propidium iodide in dead or damaged cells.

### Cathepsin activity

Supernatant from AGS cell lysates was incubated with the Ommicathepsin fluorogenic substrate (Z-FR-AMC, Enzo Life Sciences, 100 µM) for 30 min at 37 °C. Fluorescence emitted by released AMC was quantified in a plate reader (exc: 380 nm; em: 440 nm) and normalized by total protein content.

### MTT assay

Cell viability in AGS cells was detected by the Cell Proliferation Kit I (Roche, 11465007001) following the manufacturer’s instructions.

### Data and Statistical Analysis

Data are expressed as mean ± S.E.M. In some cases, data are expressed as percentage or fold-induction vs respective control values in order to obtain more illustrative graphs. However, in all cases statistical analyses were performed for absolute values. Data were compared by one way-ANOVA/repeated measures one way-ANOVA with a Newman-Keuls post hoc correction for multiple comparisons, by two way-ANOVA or by Student’s t-test when appropriate. A P value < 0.05 was considered to be statistically significant.

### Materials

Indomethacin (I7378), chloroquine (C6628), bafilomycin B1 (11707), and wortmannin (W1628) were obtained from Sigma-Aldrich. Oxaliplatin (ab141054) was obtained from Abcam. Rapamycin was kindly provided by Pfizer.

### Data availability

The authors agree to make materials, data and associated protocols promptly available to readers.

## Electronic supplementary material

Supplementary Information
