# Supplementary material for: Indomethacin Disrupts Autophagic Flux by Inducing Lysosomal Dysfunction in Gastric Cancer Cells and Increases Their Sensitivity to Cytotoxic Drugs
Source: Sci Rep. 2018 Feb 26;8:3593. doi: 10.1038/s41598-018-21455-1 (PMC5827024; doi:10.1038/s41598-018-21455-1)
Supplement: Supplementary file 1 — Supplementary Information [file 41598_2018_21455_MOESM1_ESM.pdf]

INDOMETHACIN DISRUPTS AUTOPHAGIC FLUX BY INDUCING LYSOSOMAL DYSFUNCTION IN GASTRIC CANCER CELLS AND INCREASES THEIR SENSITIVITY TO CYTOTOXIC DRUGS

Jorge Vallecillo-Hernández (jorvaher@alumni.uv.es) [1], Maria Dolores Barrachina (dolores.barrachina@uv.es) [1], Dolores Ortiz-Masiá (m.dolores.ortiz@uv.es) [2], Sandra Coll (collpuig@alumni.uv.es) [1], Juan Vicente Esplugues (juan.v.esplugues@uv.es) [1,3], Sara Calatayud [1]* (sara.calatayud@uv.es) [1], Carlos Hernández (carlos.hernandez-saez@uv.es) [1,3].

[1] Departamento de Farmacología and CIBERehd, Facultad de Medicina, Universidad de Valencia, Av. Blasco Ibáñez, 15, 46010 - Valencia, Spain.

[2] Departamento de Medicina and CIBERehd, Facultad de Medicina, Universidad de Valencia, Av. Blasco Ibáñez, 15, 46010 - Valencia, Spain.

[3] FISABIO, Hospital Dr. Peset, Av. Cataluña, 21, 46020 - Valencia, Spain.

Running title: INDOMETHACIN INHIBITS AUTOPHAGY IN CANCER CELLS

Keywords: NSAID, AUTOPHAGY, CANCER, CHEMOTHERAPY, LYSOSOMES

Correspondence to:
Sara Calatayud, PhD
Department of Pharmacology, Faculty of Medicine,
University of Valencia,
Avda.Blasco Ibáñez, 15-17
46010 Valencia (Spain)
Phone: +34 96 398 3779
Fax: +34 96 398 3778
E-mail: sara.calatayud@uv.es

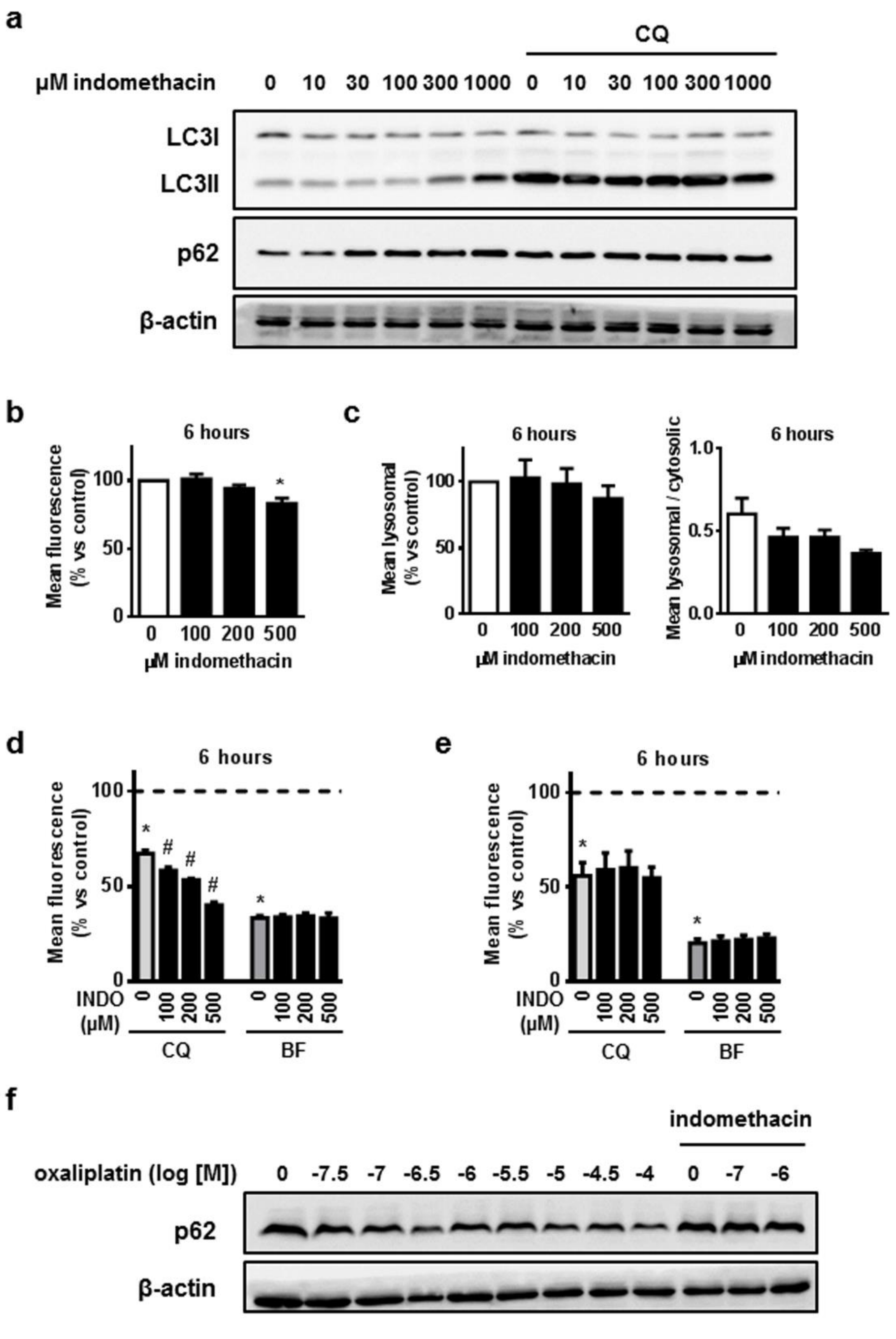

Supplementary figure S1. Indomethacin inhibits autophagy degradation in HT29 cells. **(A)** Representative Western blot for LC3, p62, and actin from cells treated with increasing doses of indomethacin in the absence and in the presence of chloroquine (20 μM) for 24 hours. **(B, D)** LysoTracker® Red DND99 fluorescence measured by static cytometry (n=3). **(C, E)** Acridine Orange red (lysosomal) fluorescence (left panels) and ratio between red

(lysosomal) / green (cytosolic) fluorescence (right panels) measured by static cytometry (n=3). **(F)** Representative Western blot for p62 and actin. In all graphs, data represent mean ± SEM. * $P<0.05$ vs. control, # $P<0.05$ vs respective inhibitor (chloroquine or bafilomycin) (ANOVA and Newman-Keuls).

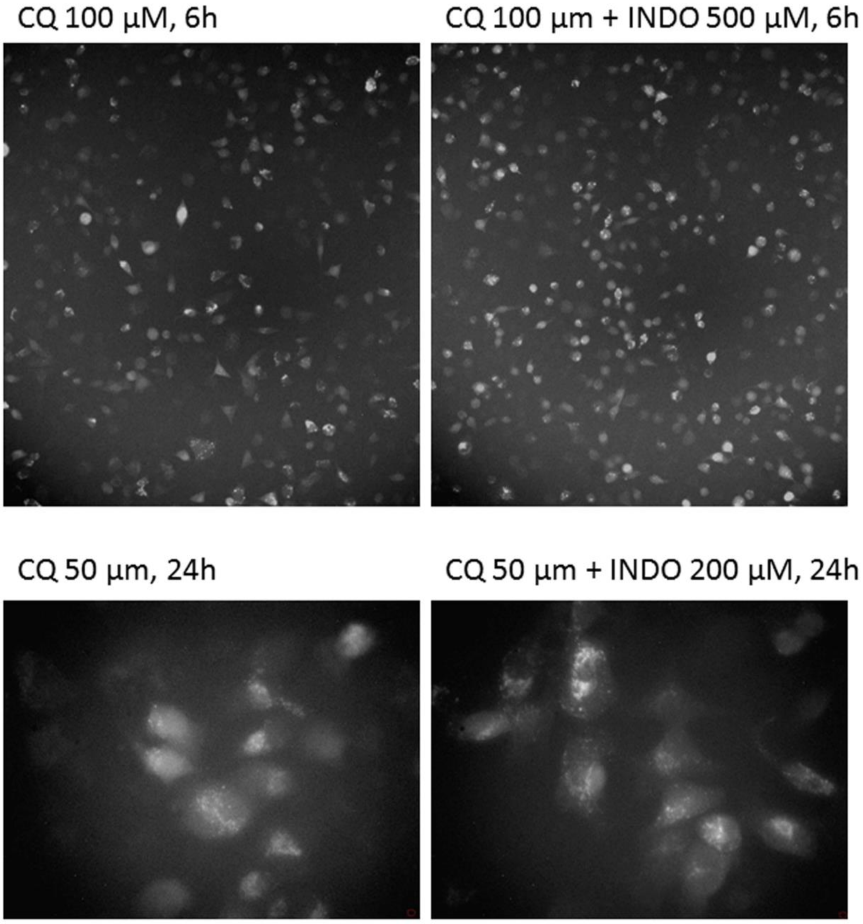

Supplementary figure S2. Representative images of LC3-II puncta in AGS cells stably expressing EmGFP-LC3B treated with chloroquine alone and combined with indomethacin. Upper panels: 10X magnification; lower panels: 40X magnification.

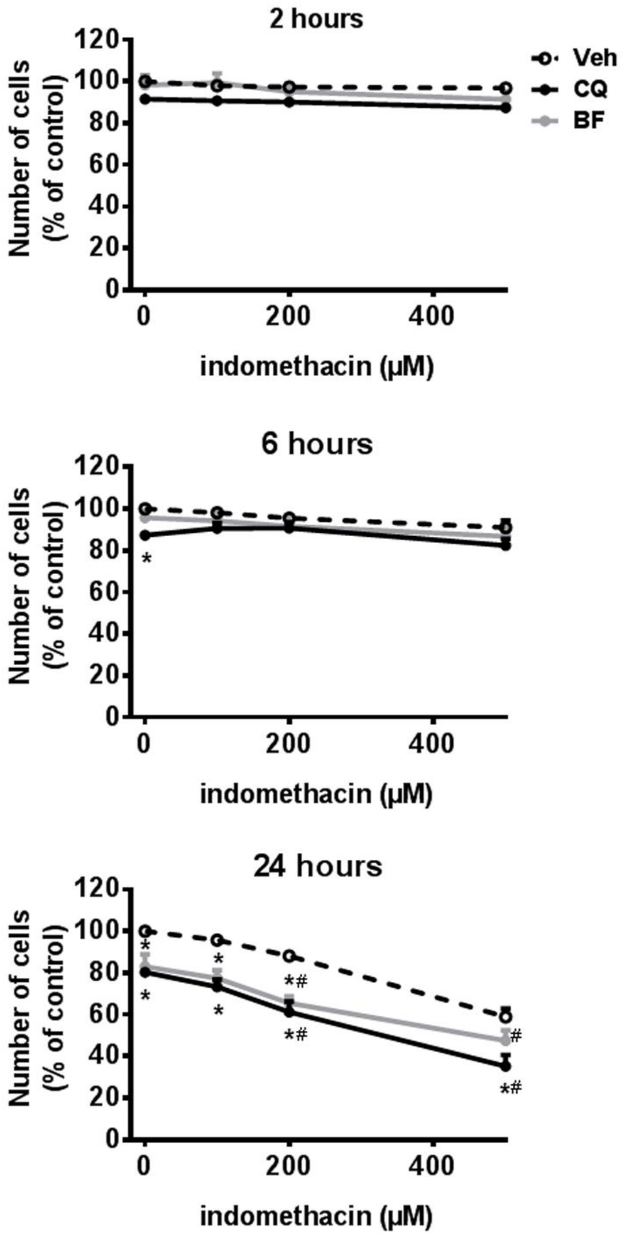

Supplementary figure S3. Effects of indomethacin, chloroquine and bafilomycin B1 in cell number in AGS cells. Cells were treated with increasing doses of indomethacin in the presence or absence of chloroquine (100 µM during 2 and 6 hours, 50 µM during 24 hours), or bafilomycin B1 (50 nM during 2, 6 and 24 hours). Cell number in each well was assessed as the number of Hoechst 33342 positive events in the fields analysed and the percentage versus the control group was calculated. Data represent mean ± SEM (n=7), * $P<0.05$ vs respective data in vehicle treated cells (ANOVA and Newman-Keuls).

Supplementary table S1. Antibodies used in immunocytochemistry (ICC) and western blot (WB) studies.

**Primary antibody**	**Technique**	**Dilution**
Rabbit polyclonal anti-LC3 (Sigma, L8918)	WB	1:1000
Mouse monoclonal anti-p62 (Santa Cruz, sc28359)	WB	1:1000
Rabbit polyclonal anti-NBR1 (Proteintech, 16004-1-AP)	WB	1:1000
Rabbit polyclonal anti-phospho-mTOR (Ser 2481) (Cell Signaling, 2974)	WB	1:1000
Rabbit polyclonal anti-mTOR (Cell Signaling, 2983)	WB	1:1000
Rabbit polyclonal anti-Actin (Sigma, A5060)	WB	1:1000
Mouse monoclonal anti-LAMP2 (Santa Cruz, sc18822)	ICC	1:100
**Secondary antibody**	**Technique**	**Dilution**
HRP-conjugated anti-mouse IgG (DakoCytomation, P0260)	WB	1:2000
HRP-conjugated anti-rabbit IgG (Vector, PI1000)	WB	1:5000
TR-conjugated goat anti-mouse IgG, F(ab')2 (Santa Cruz, sc3779)	ICC	1:200
